# Diphenyl(pyridin-2-yl)­phosphane selenide

**DOI:** 10.1107/S160053681204007X

**Published:** 2012-10-06

**Authors:** Wade L. Davis, Alfred Muller

**Affiliations:** a Research Centre for Synthesis and Catalysis, Department of Chemistry, University of Johannesburg (APK Campus), PO Box 524, Auckland Park, Johannesburg, 2006, South Africa

## Abstract

In the title compound, C_17_H_14_NPSe, the P atom has a distorted tetra­hedral environment resulting in an effective cone angle of 163°. In the crystal, C—H⋯Se/N/π inter­actions are observed.

## Related literature

For background to phospho­rus- and selenium-containing ligands, see: Muller *et al.* (2006 ▶, 2008 ▶). For the free phosphine of the title compound, see: Charland *et al.* (1989 ▶). For background on cone angles, see: Otto (2001 ▶); Tolman (1977 ▶). For details of the conformational fit of the two mol­ecules using *Mercury*, see: Macrae *et al.* (2008 ▶); Weng *et al.* (2008*a* ▶,*b* ▶).
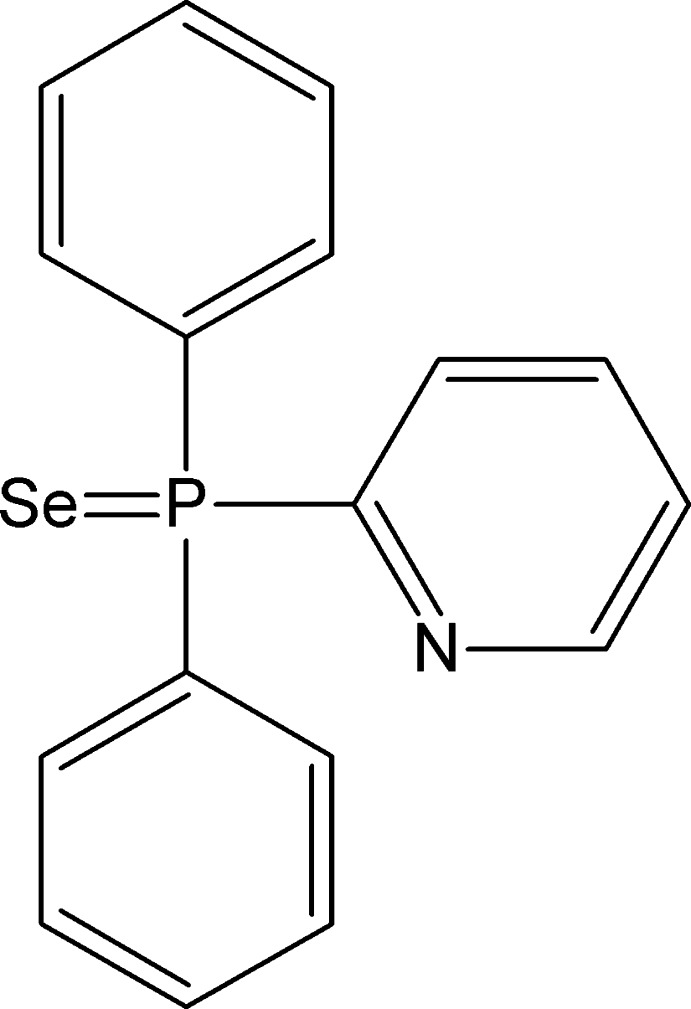


## Experimental

### 

#### Crystal data

C_17_H_14_NPSe
*M* *_r_* = 342.22
Orthorhombic, 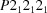

*a* = 8.8092 (4) Å
*b* = 9.4066 (4) Å
*c* = 18.2661 (7) Å
*V* = 1513.61 (11) Å^3^
*Z* = 4
Cu *K*α radiation
μ = 4.25 mm^−1^
*T* = 100 K
0.24 × 0.17 × 0.12 mm

#### Data collection

Bruker APEX DUO 4K-CCD diffractometer
Absorption correction: multi-scan (*SADABS*; Bruker, 2008 ▶) *T*_min_ = 0.429, *T*_max_ = 0.629
6004 measured reflections
2501 independent reflections
2461 reflections with *I* > 2σ(*I*)
*R*_int_ = 0.025

#### Refinement

*R*[*F* ^2^ > 2σ(*F* ^2^)] = 0.021
*wR*(*F* ^2^) = 0.053
*S* = 0.87
2501 reflections
181 parameters
H-atom parameters constrained
Δρ_max_ = 0.43 e Å^−3^
Δρ_min_ = −0.27 e Å^−3^
Absolute structure: Flack (1983 ▶), with 992 Friedel pairs
Flack parameter: 0.053 (19)

### 

Data collection: *APEX2* (Bruker, 2011 ▶); cell refinement: *SAINT* (Bruker, 2008 ▶); data reduction: *SAINT* and *XPREP* (Bruker, 2008 ▶); program(s) used to solve structure: *SIR97* (Altomare *et al.*, 1999 ▶); program(s) used to refine structure: *SHELXL97* (Sheldrick, 2008 ▶); molecular graphics: *DIAMOND* (Brandenburg & Putz, 2005 ▶); software used to prepare material for publication: *publCIF* (Westrip, 2010 ▶) and *WinGX* (Farrugia, 1999 ▶).

## Supplementary Material

Crystal structure: contains datablock(s) global, I. DOI: 10.1107/S160053681204007X/kp2438sup1.cif

Structure factors: contains datablock(s) I. DOI: 10.1107/S160053681204007X/kp2438Isup2.hkl

Supplementary material file. DOI: 10.1107/S160053681204007X/kp2438Isup3.cml

Additional supplementary materials:  crystallographic information; 3D view; checkCIF report

## Figures and Tables

**Table 1:** Hydrogen-bond geometry (Å, °) *Cg*1 is the centroid of the C1–C6 ring.

*D*—H⋯*A*	*D*—H	H⋯*A*	*D*⋯*A*	*D*—H⋯*A*
C12—H12⋯Se1	0.95	2.87	3.427 (3)	118
C8—H8⋯N1	0.95	2.57	3.111 (3)	116
C14—H14⋯Se1	0.95	2.96	3.472 (2)	115
C5—H5⋯Se1^i^	0.95	3.07	3.923 (3)	150
C16—H16⋯Se1^ii^	0.95	3.26	3.938 (3)	130
C11—H11⋯*Cg*1^iii^	0.95	2.77	3.630 (3)	151

Symmetry codes: (i) 
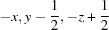
; (ii) 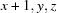; (iii) 
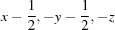
.

**Fig. 1.**
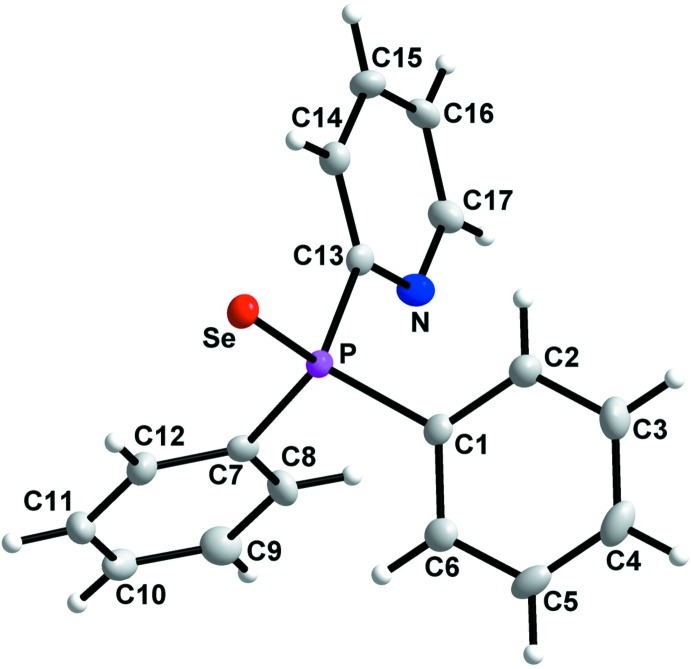
A view of (1). Displacement ellipsoids are drawn at the 50% probability level.

**Fig. 2.**
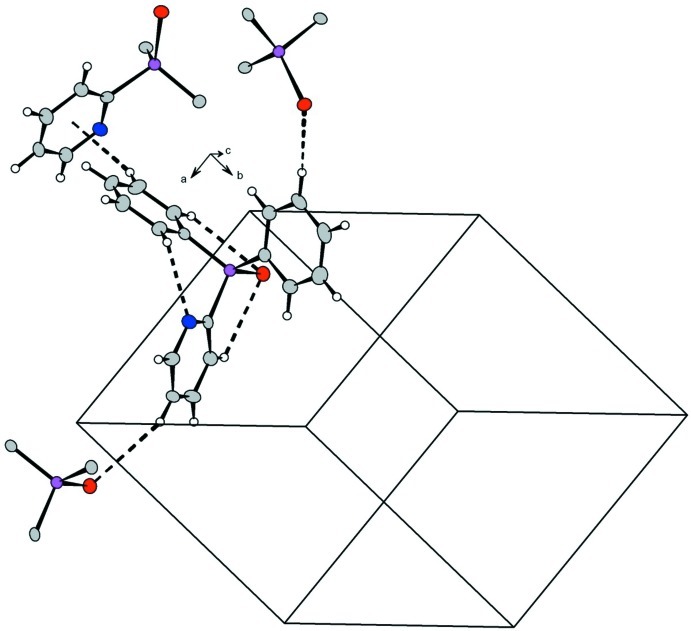
Packing diagram of (1) showing the C—H···Se/N/π interactions.

**Fig. 3.**
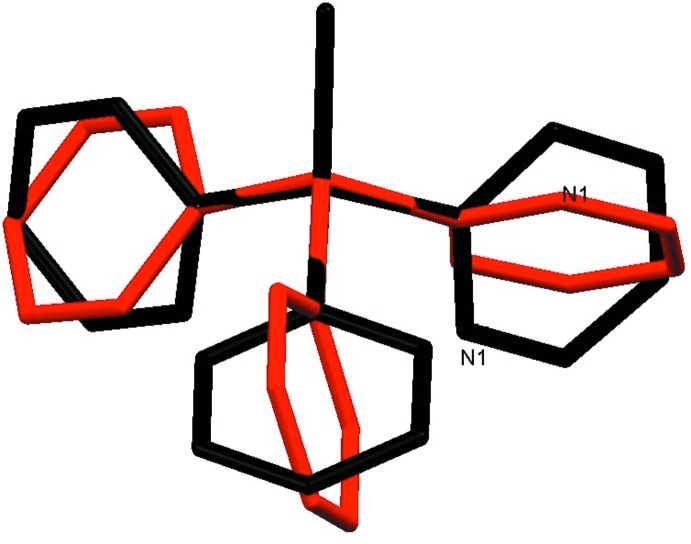
Conformational similarity between the title compound (black) and the free phosphine (red).

## supplementary crystallographic information

### Comment

As part our systematic investigation on the steric and electronic properties of phosphorus containing ligands, we are also utilizing the ^1^*J*(^31^P-^77^Se) multi-nuclear NMR coupling in Se—P bond as a probe (see Muller *et al.*, 2008). The advantage of this approach is that there is no steric crowding effect, albeit crystal packing effects, as normally found in transition metal complexes with bulky ligands, *e.g.* in *trans*-[Rh(CO)Cl{P(OC_6_H_5_)_3_}_2_] cone angles variation from 156° to 167° was observed for the two phosphite ligands (Muller *et al.*, 2006). Herein we report here the single-crystal structure of SePPh_2_py, where Ph = C_6_H_5_ and py = C_5_H_4_N as part of our investigation.

Molecules of the title compound (Fig. 1) adopts a distorted tetrahedral arrangement about the P atom with average C—P—C and Se—P—C angles of 105.47° and 113.20° respectively. Describing the steric demand of phosphane ligands has been the topic of many studies and a variety of models have been developed. The Tolman cone angle (Tolman, 1977) is still the most commonly used model. Applying this model to the geometry obtained for the title compound (and adjusting the Se—P bond distance to 2.28 Å) we calculated an effective cone angle from the geometry found in the crystal structure of 163° (Otto, 2001). The angle calculated is 9° larger than that of the free phosphine (Charland *et al.*, 1989; effective cone angle calculated as 154°), and could be ascribed to C—H···Se/N/π intra- and interactions observed in the title compound (Table 1, Fig. 2), whereas the free phosphine shows C—H···N/π interactions only. The difference in the orientation of the substituents for these two structures can be illustrated by superimposing their coordinates (Fig. 3); root mean squared deviation calculated as 0.0468 Å for P and *ipso* C atoms only using *Mercury* (Macrae *et al.*, 2008; Weng *et al.*, 2008*a*,*b*).

### Experimental

Diphenyl-2-pyridylphosphine and KSeCN were purchased from Sigma–Aldrich and used without purification. Eqimolar amounts of KSeCN (5.8 mg, 0.04 mmol) and the diphenylpyridylphosphine (10.5 mg, 0.04 mmol) were dissolved in the minimum amounts of methanol (10 ml). The KSeCN solution was added dropwise (5 min) to the phosphine solution with stirring at room temperature. The final solution was left to evaporate slowly until dry to give crystals suitable for a single-crystal X-ray study. Analytical data: ^31^P {H} NMR (CDCl_3_, 161.99 MHz): δ = 31.47 (t, ^1^*J*(^31^P-^77^Se) = 734 Hz).

### Refinement

The aromatic H atoms were placed in geometrically idealized positions with C—H = 0.95 Å, and allowed to ride on their parent atoms, with *U*_iso_(H) = 1.2*U*_eq_(C).

### Crystal data

**Table:**

C_17_H_14_NPSe	*F*(000) = 688
*M**_r_* = 342.22	*D*_x_ = 1.502 Mg m^−^^3^
Orthorhombic, *P*2_1_2_1_2_1_	Cu *K*α radiation, λ = 1.54178 Å
Hall symbol: P 2ac 2ab	Cell parameters from 5093 reflections
*a* = 8.8092 (4) Å	θ = 4.8–66.6°
*b* = 9.4066 (4) Å	µ = 4.25 mm^−^^1^
*c* = 18.2661 (7) Å	*T* = 100 K
*V* = 1513.61 (11) Å^3^	Cuboid, colourless
*Z* = 4	0.24 × 0.17 × 0.12 mm

### Data collection

**Table:**

Bruker APEX DUO 4K-CCD diffractometer	2501 independent reflections
Incoatec Quazar Multilayer Mirror monochromator	2461 reflections with *I* > 2σ(*I*)
Detector resolution: 8.4 pixels mm^-1^	*R*_int_ = 0.025
φ and ω scans	θ_max_ = 66.6°, θ_min_ = 4.8°
Absorption correction: multi-scan (*SADABS*; Bruker, 2008)	*h* = −9→10
*T*_min_ = 0.429, *T*_max_ = 0.629	*k* = −3→11
6004 measured reflections	*l* = −21→20

### Refinement

**Table:**

Refinement on *F*^2^	Secondary atom site location: difference Fourier map
Least-squares matrix: full	Hydrogen site location: inferred from neighbouring sites
*R*[*F*^2^ > 2σ(*F*^2^)] = 0.021	H-atom parameters constrained
*wR*(*F*^2^) = 0.053	*w* = 1/[σ^2^(*F*_o_^2^) + (0.0288*P*)^2^ + 1.1995*P*] where *P* = (*F*_o_^2^ + 2*F*_c_^2^)/3
*S* = 0.87	(Δ/σ)_max_ < 0.001
2501 reflections	Δρ_max_ = 0.43 e Å^−^^3^
181 parameters	Δρ_min_ = −0.27 e Å^−^^3^
0 restraints	Absolute structure: Flack (1983), with 992 Friedel pairs
Primary atom site location: structure-invariant direct methods	Flack parameter: 0.053 (19)

### Special details

**Table:**

Experimental. The intensity data was collected on a Bruker Apex DUO 4 K CCD diffractometer using an exposure time of 5 s/frame. A total of 287 frames were collected with a frame width of 4° covering up to θ = 66.62° with 96.7% completeness accomplished.
Geometry. All e.s.d.'s (except the e.s.d. in the dihedral angle between two l.s. planes) are estimated using the full covariance matrix. The cell e.s.d.'s are taken into account individually in the estimation of e.s.d.'s in distances, angles and torsion angles; correlations between e.s.d.'s in cell parameters are only used when they are defined by crystal symmetry. An approximate (isotropic) treatment of cell e.s.d.'s is used for estimating e.s.d.'s involving l.s. planes.
Refinement. Refinement of *F*^2^ against ALL reflections. The weighted *R*-factor *wR* and goodness of fit *S* are based on *F*^2^, conventional *R*-factors *R* are based on *F*, with *F* set to zero for negative *F*^2^. The threshold expression of *F*^2^ > σ(*F*^2^) is used only for calculating *R*-factors(gt) *etc*. and is not relevant to the choice of reflections for refinement. *R*-factors based on *F*^2^ are statistically about twice as large as those based on *F*, and *R*-factors based on ALL data will be even larger.

### Fractional atomic coordinates and isotropic or equivalent isotropic displacement parameters (Å^2^)

**Table:**

	*x*	*y*	*z*	*U*_iso_*/*U*_eq_	
Se1	0.16691 (3)	0.13803 (3)	0.059243 (13)	0.01846 (8)	
P1	0.28195 (7)	−0.00265 (7)	0.12974 (3)	0.01351 (13)	
N1	0.5564 (3)	−0.0376 (2)	0.19118 (12)	0.0211 (5)	
C1	0.2066 (3)	−0.0007 (3)	0.22196 (12)	0.0155 (5)	
C2	0.2524 (3)	0.1080 (3)	0.26909 (13)	0.0222 (6)	
H2	0.3282	0.1737	0.2541	0.027*	
C3	0.1867 (3)	0.1197 (3)	0.33781 (14)	0.0279 (6)	
H3	0.2185	0.1927	0.3703	0.034*	
C4	0.0749 (3)	0.0250 (3)	0.35923 (14)	0.0271 (6)	
H4	0.0292	0.0341	0.4061	0.033*	
C5	0.0295 (3)	−0.0826 (3)	0.31256 (14)	0.0226 (6)	
H5	−0.0465	−0.1479	0.3276	0.027*	
C6	0.0953 (3)	−0.0952 (3)	0.24349 (14)	0.0192 (6)	
H6	0.0636	−0.1687	0.2113	0.023*	
C7	0.2834 (3)	−0.1863 (3)	0.09839 (13)	0.0165 (5)	
C12	0.2168 (3)	−0.2192 (3)	0.03143 (13)	0.0218 (6)	
H12	0.1654	−0.1481	0.0041	0.026*	
C11	0.2266 (3)	−0.3580 (3)	0.00496 (13)	0.0257 (6)	
H11	0.1802	−0.382	−0.0403	0.031*	
C10	0.3034 (3)	−0.4608 (3)	0.04429 (14)	0.0249 (6)	
H10	0.3129	−0.5543	0.0252	0.03*	
C9	0.3666 (3)	−0.4276 (3)	0.11135 (15)	0.0271 (6)	
H9	0.417	−0.499	0.1389	0.033*	
C8	0.3567 (3)	−0.2906 (3)	0.13858 (14)	0.0216 (6)	
H8	0.4001	−0.2681	0.1848	0.026*	
C13	0.4816 (3)	0.0441 (3)	0.14240 (13)	0.0153 (5)	
C17	0.7046 (3)	−0.0092 (3)	0.20170 (14)	0.0222 (6)	
H17	0.7597	−0.0657	0.2356	0.027*	
C16	0.7802 (3)	0.0985 (3)	0.16541 (14)	0.0203 (6)	
H16	0.8851	0.1146	0.1741	0.024*	
C15	0.7021 (3)	0.1817 (3)	0.11670 (14)	0.0221 (6)	
H15	0.7518	0.2571	0.0917	0.026*	
C14	0.5494 (3)	0.1544 (3)	0.10432 (13)	0.0193 (5)	
H14	0.4928	0.21	0.0706	0.023*	

### Atomic displacement parameters (Å^2^)

**Table:**

	*U*^11^	*U*^22^	*U*^33^	*U*^12^	*U*^13^	*U*^23^
Se1	0.01952 (13)	0.01885 (13)	0.01701 (12)	0.00321 (11)	−0.00156 (10)	0.00276 (10)
P1	0.0138 (3)	0.0140 (3)	0.0127 (3)	0.0002 (3)	0.0005 (2)	0.0005 (2)
N1	0.0168 (11)	0.0207 (12)	0.0257 (11)	0.0009 (9)	−0.0010 (9)	0.0027 (9)
C1	0.0160 (13)	0.0164 (12)	0.0142 (10)	0.0031 (11)	−0.0007 (9)	0.0023 (9)
C2	0.0217 (14)	0.0247 (16)	0.0201 (13)	−0.0027 (11)	0.0031 (10)	−0.0015 (10)
C3	0.0336 (16)	0.0306 (15)	0.0196 (13)	0.0013 (15)	0.0005 (11)	−0.0057 (11)
C4	0.0285 (16)	0.0351 (17)	0.0178 (13)	0.0114 (13)	0.0059 (11)	0.0033 (11)
C5	0.0170 (13)	0.0280 (15)	0.0229 (13)	0.0036 (11)	0.0054 (11)	0.0099 (11)
C6	0.0185 (13)	0.0200 (15)	0.0193 (12)	0.0024 (11)	−0.0020 (10)	0.0028 (10)
C7	0.0141 (12)	0.0161 (13)	0.0194 (12)	−0.0012 (10)	0.0058 (10)	−0.0019 (9)
C12	0.0256 (13)	0.0230 (14)	0.0167 (12)	−0.0026 (12)	0.0021 (10)	0.0000 (10)
C11	0.0360 (14)	0.0233 (13)	0.0176 (12)	−0.0096 (14)	0.0010 (11)	−0.0032 (11)
C10	0.0329 (16)	0.0172 (13)	0.0246 (13)	−0.0034 (11)	0.0103 (11)	−0.0040 (10)
C9	0.0273 (17)	0.0213 (14)	0.0327 (15)	0.0037 (12)	0.0009 (12)	0.0000 (11)
C8	0.0216 (14)	0.0233 (13)	0.0199 (12)	−0.0015 (12)	−0.0043 (11)	−0.0036 (10)
C13	0.0142 (12)	0.0168 (13)	0.0149 (11)	0.0028 (10)	0.0030 (9)	−0.0026 (9)
C17	0.0210 (14)	0.0206 (13)	0.0250 (13)	−0.0015 (13)	−0.0041 (10)	0.0007 (11)
C16	0.0140 (12)	0.0222 (15)	0.0248 (13)	−0.0042 (11)	0.0014 (10)	−0.0057 (10)
C15	0.0191 (14)	0.0244 (14)	0.0227 (12)	−0.0061 (11)	0.0025 (10)	0.0026 (10)
C14	0.0185 (12)	0.0213 (14)	0.0179 (12)	0.0000 (12)	−0.0007 (9)	0.0002 (10)

### Geometric parameters (Å, º)

**Table:**

Se1—P1	2.1063 (6)	C7—C12	1.391 (3)
P1—C1	1.811 (2)	C12—C11	1.395 (4)
P1—C7	1.820 (2)	C12—H12	0.95
P1—C13	1.828 (3)	C11—C10	1.381 (4)
N1—C13	1.349 (3)	C11—H11	0.95
N1—C17	1.347 (3)	C10—C9	1.381 (4)
C1—C6	1.380 (4)	C10—H10	0.95
C1—C2	1.397 (4)	C9—C8	1.384 (4)
C2—C3	1.387 (4)	C9—H9	0.95
C2—H2	0.95	C8—H8	0.95
C3—C4	1.384 (4)	C13—C14	1.384 (4)
C3—H3	0.95	C17—C16	1.381 (4)
C4—C5	1.382 (4)	C17—H17	0.95
C4—H4	0.95	C16—C15	1.370 (4)
C5—C6	1.393 (4)	C16—H16	0.95
C5—H5	0.95	C15—C14	1.388 (4)
C6—H6	0.95	C15—H15	0.95
C7—C8	1.385 (4)	C14—H14	0.95
			
C1—P1—C7	107.75 (11)	C7—C12—H12	120.4
C1—P1—C13	103.46 (11)	C11—C12—H12	120.4
C7—P1—C13	105.15 (11)	C10—C11—C12	120.3 (2)
C1—P1—Se1	112.71 (8)	C10—C11—H11	119.8
C7—P1—Se1	114.04 (9)	C12—C11—H11	119.8
C13—P1—Se1	112.90 (8)	C11—C10—C9	120.0 (2)
C13—N1—C17	117.0 (2)	C11—C10—H10	120
C6—C1—C2	120.1 (2)	C9—C10—H10	120
C6—C1—P1	121.27 (19)	C10—C9—C8	120.3 (3)
C2—C1—P1	118.34 (19)	C10—C9—H9	119.9
C3—C2—C1	119.7 (2)	C8—C9—H9	119.9
C3—C2—H2	120.2	C7—C8—C9	119.9 (2)
C1—C2—H2	120.2	C7—C8—H8	120.1
C4—C3—C2	120.2 (3)	C9—C8—H8	120.1
C4—C3—H3	119.9	N1—C13—C14	123.3 (2)
C2—C3—H3	119.9	N1—C13—P1	114.59 (18)
C3—C4—C5	120.1 (2)	C14—C13—P1	122.12 (19)
C3—C4—H4	119.9	N1—C17—C16	123.0 (2)
C5—C4—H4	119.9	N1—C17—H17	118.5
C4—C5—C6	120.1 (3)	C16—C17—H17	118.5
C4—C5—H5	120	C15—C16—C17	119.2 (2)
C6—C5—H5	120	C15—C16—H16	120.4
C1—C6—C5	119.9 (2)	C17—C16—H16	120.4
C1—C6—H6	120.1	C16—C15—C14	119.1 (2)
C5—C6—H6	120.1	C16—C15—H15	120.4
C8—C7—C12	120.3 (2)	C14—C15—H15	120.4
C8—C7—P1	120.58 (19)	C13—C14—C15	118.3 (2)
C12—C7—P1	119.0 (2)	C13—C14—H14	120.8
C7—C12—C11	119.1 (3)	C15—C14—H14	120.8
			
C7—P1—C1—C6	−34.1 (2)	P1—C7—C12—C11	175.8 (2)
C13—P1—C1—C6	−145.1 (2)	C7—C12—C11—C10	−1.0 (4)
Se1—P1—C1—C6	92.6 (2)	C12—C11—C10—C9	2.2 (4)
C7—P1—C1—C2	152.3 (2)	C11—C10—C9—C8	−1.7 (4)
C13—P1—C1—C2	41.3 (2)	C12—C7—C8—C9	1.4 (4)
Se1—P1—C1—C2	−81.0 (2)	P1—C7—C8—C9	−175.2 (2)
C6—C1—C2—C3	0.7 (4)	C10—C9—C8—C7	−0.1 (4)
P1—C1—C2—C3	174.4 (2)	C17—N1—C13—C14	−0.7 (4)
C1—C2—C3—C4	−0.8 (4)	C17—N1—C13—P1	178.94 (19)
C2—C3—C4—C5	0.8 (4)	C1—P1—C13—N1	53.4 (2)
C3—C4—C5—C6	−0.6 (4)	C7—P1—C13—N1	−59.5 (2)
C2—C1—C6—C5	−0.5 (4)	Se1—P1—C13—N1	175.59 (16)
P1—C1—C6—C5	−174.0 (2)	C1—P1—C13—C14	−126.9 (2)
C4—C5—C6—C1	0.5 (4)	C7—P1—C13—C14	120.2 (2)
C1—P1—C7—C8	−55.0 (2)	Se1—P1—C13—C14	−4.7 (2)
C13—P1—C7—C8	54.9 (2)	C13—N1—C17—C16	0.3 (4)
Se1—P1—C7—C8	179.09 (18)	N1—C17—C16—C15	0.5 (4)
C1—P1—C7—C12	128.4 (2)	C17—C16—C15—C14	−0.9 (4)
C13—P1—C7—C12	−121.7 (2)	N1—C13—C14—C15	0.4 (4)
Se1—P1—C7—C12	2.5 (2)	P1—C13—C14—C15	−179.30 (19)
C8—C7—C12—C11	−0.8 (4)	C16—C15—C14—C13	0.5 (4)

### Hydrogen-bond geometry (Å, º)

Cg1 is the centroid of the C1–C6 ring.

**Table:**

*D*—H···*A*	*D*—H	H···*A*	*D*···*A*	*D*—H···*A*
C12—H12···Se1	0.95	2.87	3.427 (3)	118
C8—H8···N1	0.95	2.57	3.111 (3)	116
C14—H14···Se1	0.95	2.96	3.472 (2)	115
C5—H5···Se1^i^	0.95	3.07	3.923 (3)	150
C16—H16···Se1^ii^	0.95	3.26	3.938 (3)	130
C11—H11···*Cg*1^iii^	0.95	2.77	3.630 (3)	151

Symmetry codes: (i) −*x*, *y*−1/2, −*z*+1/2; (ii) *x*+1, *y*, *z*; (iii) *x*−1/2, −*y*−1/2, −*z*.
